# Delirium, Sedation and Analgesia in the Intensive Care Unit: A Multinational, Two-Part Survey among Intensivists

**DOI:** 10.1371/journal.pone.0110935

**Published:** 2014-11-14

**Authors:** Alawi Luetz, Felix Balzer, Finn M. Radtke, Christina Jones, Giuseppe Citerio, Bernhard Walder, Bjoern Weiss, Klaus-Dieter Wernecke, Claudia Spies

**Affiliations:** 1 Department of Anesthesiology and Intensive Care Medicine, Campus Charité Mitte and Campus Virchow-Klinikum, Charité – Universitätsmedizin Berlin, Berlin, Germany; 2 Whiston Hospital, Critical Care Unit, Prescot, Liverpool, United Kingdom; 3 Hospital San Gerardo, Department of Emergency Medicine, Monza, Italy; 4 Service d'Anesthesiologie, Hôpitaux Universitaires de Genève, Geneva, Switzerland; 5 Sostana GmbH, Berlin, Germany; Catholic University of Sacred Heart of Rome, Italy

## Abstract

Analgesia, sedation and delirium management are important parts of intensive care treatment as they are relevant for patients' clinical and functional long-term outcome. Previous surveys showed that despite this fact implementation rates are still low. The primary aim of the prospective, observational multicenter study was to investigate the implementation rate of delirium monitoring among intensivists. Secondly, current practice concerning analgesia and sedation monitoring as well as treatment strategies for patients with delirium were assesed. In addition, this study compares perceived and actual practice regarding delirium, sedation and analgesia management. Data were obtained with a two-part, anonymous survey, containing general data from intensive care units in a first part and data referring to individual patients in a second part. Questionnaires from 101 hospitals (part 1) and 868 patients (part 2) were included in data analysis. Fifty-six percent of the intensive care units reported to monitor for delirium in clinical routine. Fourty-four percent reported the use of a validated delirium score. In this respect, the survey suggests an increasing use of delirium assessment tools compared to previous surveys. Nevertheless, part two of the survey revealed that in actual practice 73% of included patients were not monitored with a validated score. Furthermore, we observed a trend towards moderate or deep sedation which is contradicting to guideline-recommendations. Every fifth patient was suffering from pain. The implementation rate of adequate pain-assessment tools for mechanically ventilated and sedated patients was low (30%). In conclusion, further efforts are necessary to implement guideline recommendations into clinical practice. The study was registered (ClinicalTrials.gov identifier: NCT01278524) and approved by the ethical committee.

## Introduction

The management of pain, sedation and delirium has a significant impact on patients' clinical and functional long-term outcome.

Delirium affects up to 82% of the critically ill patients and is associated with long-term cognitive impairment [1] and a 3-fold increase of 6-month mortality [2]. Studies revealed that intensive care unit (ICU) delirium is underrecognized by intensivists and nurses in daily routine care [3]. Using a validated assessment tool significantly improves the ability of physicians [4] and nurses [5] to identify ICU delirium.

Sedation practice predicts long-term mortality in critically ill patients [6] and requires monitoring to define adequate targets and control the effect of applied sedatives.

Pain is the major stressor for critically ill patients [7] and chronic pain is a severe complication that was reported by 44% of patients 6 months to 1 year after ICU discharge [8]. Assessment for pain in mechanically ventilated patients is independently associated with improved outcome [9].

On a national as well as international level, societies of critical and intensive care medicine have taken efforts such as supporting the development of guidelines [10]–[12] and offering simulation- as well as online-training tools to drive attention on analgesia, sedation and delirium management. Nevertheless, previous national and international surveys demonstrated a low implementation of these screening tools into clinical practice: e.g., a survey conducted on 1384 ICU practitioners in North America revealed that more than half of them (59%) assessed their patients for delirium but only 20% used a valid delirium assessment tool [13].

The primary aim of this prospective, observational multicenter study was to investigate the implementation rate of delirium monitoring among intensivists. Secondly, we assessed the current practice of analgesia and sedation monitoring as well as treatment strategies for delirium. Finally, this study compares perceived and actual practice regarding delirium, sedation and analgesia management.

## Results

### Part one - Hospital and ICU data

**Figure 1:**
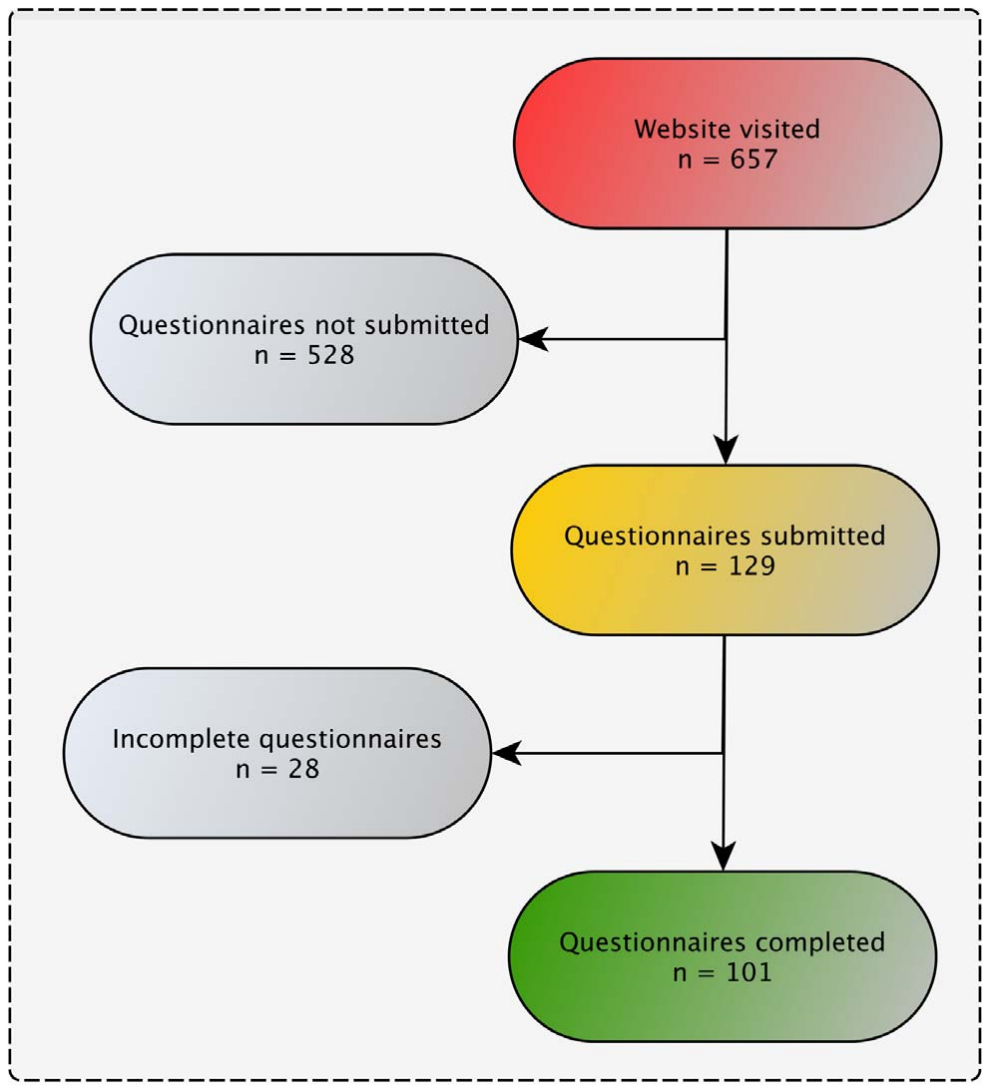
Consort diagram for questionnaire part one. This part of the survey gathered general information about the participating hospitals as well as (non-)pharmacological strategies for the management of analgesia, delirium and sedation.

The first questionnaire ("part one") yielded 101 complete forms that were included in the data analysis. Five hundred and fifyt-six forms were either not submitted or not completed (figure 1). Characteristics of the ICUs that participated in the survey are presented in detail in table 1. The median of patients per physician was 4 (3–6), and the median number of patients per registered nurse was 2 (1–2). The median number of beds per ICU was 12 (8–18).

**Table 1:** Characteristics of participating intensive care units.

Characteristics	n (%)
Type of hospital	
University	56 (55)
Teaching	31 (31)
Other	14 (14)
Type of intensive care unit	
Surgical	26 (26)
Medical	5 (5)
Mixed	69 (69)
Equipped for invasive mechanical ventilation	
All beds	75 (74)
Some beds	24 (24)
No beds	2 (2)
Estimated mean intensive care unit length of stay	
1–3 days	14 (14)
3–7 days	68 (67)
>8 days	15 (15)
No answer	4 (4)

Fifty-six percent (n = 56) of all ICUs reported some kind of screening for symptoms of delirium (table 2). Fourty-four percent (n = 44) used a validated delirium screening tool. The most frequently used score was the Confusion Assessment Method for the ICU (CAM-ICU) (n = 37, 84%) followed by the Intensive Care Delirium Screening Checklist (ICDSC) (n = 3, 7%), the Nursing Delirium Screening Scale (Nu-DESC) (n = 2, 5%), the Delirium Detection Score (DDS) (n = 1, 2%) and the Diagnostic and Statistical Manual of Mental Disorders, Version IV (n = 1, 2%).

**Table 2:** Frequencies of analgesia, delirium and sedation monitoring in participating intensive care units.

Frequency	Analgesia	Delirium	Sedation
	n (%)	n (%)	n (%)
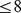 hours	50 (49)	30 (30)	79 (78)
>8 hours	9 (9)	13 (13)	5 (5)
Daily	1 (1)	13 (13)	5 (5)
As needed	21 (21)	0 (0)	0 (0)
Never	20 (20)	45 (44)	12 (12)

Reported frequencies include validated as well as non-validated methods.

Routine sedation monitoring was implemented in 88% (n = 89) of the ICUs (table 2). The most frequently used tool was the Richmond Agitation Sedation Scale (RASS) (n = 48, 54%). The Ramsay Sedation Scale was used by 27% (n = 24) and the Sedation Agitation Scale (SAS) by 6% (n = 5) of the participating ICUs. Thirteen percent (n = 12) of the respondents performed sedation monitoring without the use of a sedation score. Thirty-nine percent (n = 39) of the ICUs performed daily spontaneous breathing trials (SBT) and 44% (n = 44) conducted daily spontaneous awakening trials (SAT). The implementation rate of a daily, paired SBT and SAT was 34% (n = 34).

Eighty percent (n = 81) of the ICUs did routinely monitor for pain (table 2). Ninety-three percent (n = 72) of them used a validated tool for pain assessment. Thirty percent (n = 24) made use of an instrument which was validated for deeply sedated and mechanically ventilated patients. The most frequently used pain score was the Visual Analogue Scale (VAS) (63%, n = 45) followed by the Numeric Rating Scale (NRS) (57%, n = 41).

Almost all ICUs (98%) treated delirious patients with specific pharmacological agents. Antipsychotics (APDs) were the most frequently used agents (99%, n = 98). Eighty-two percent (n = 81) of the ICUs used benzodiazepines (BDZs) as a part of their treatment regime table 3.

**Table 3:** Pharmacological treatment strategies for delirium as applied by the participating intensive care units.

Drug type	n (%)
Exclusively APDs	6 (6)
Exclusively BDZs	1 (1)
APDs and BDZs	18 (18)
APDs and Other*	12 (12)
APDs, BDZs and Other*	62 (61)
No answer	2 (2)

APDs, antipsychotics (e.g. haloperidol). BDZs, benzodiazepines. *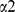 adrenergic agonist, selective serotonin re-uptake inhibitors.

**Figure 2:**
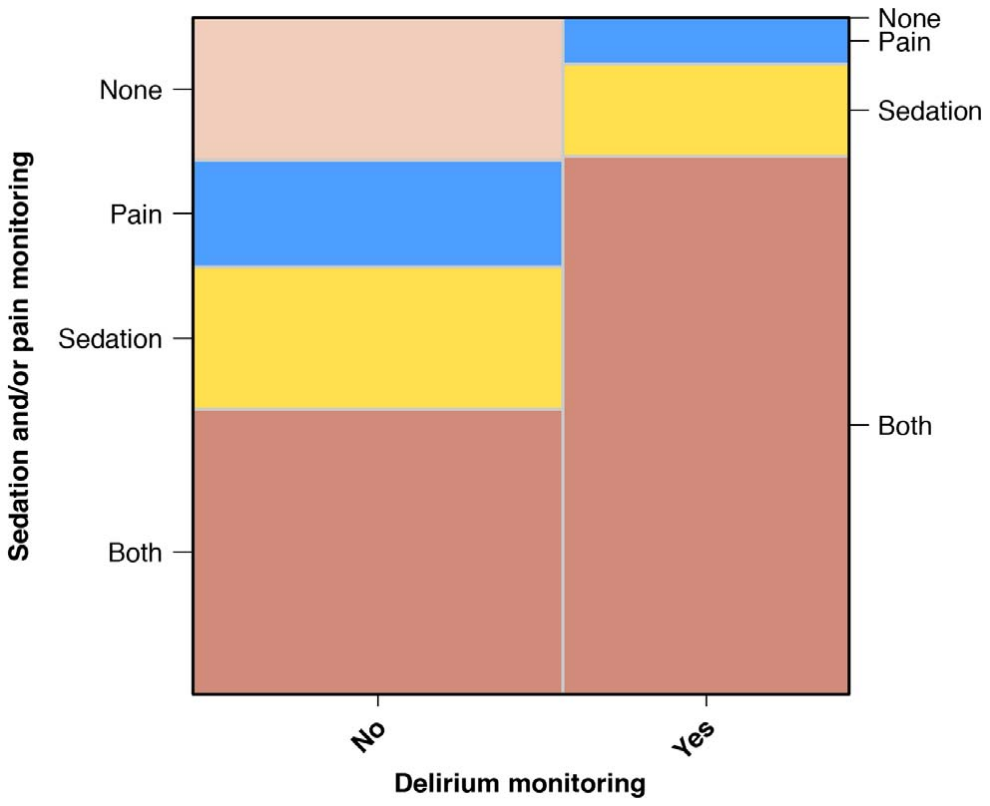
Monitoring of sedation, analgesia and delirium with validated scores. Mosaic plot: The areas of the mosaic tiles are proportional to the observed frequency of groups. Both, sedation and pain monitoring. None, no sedation and no pain monitoring.

ICUs which performed routine delirium monitoring with a validated tool had implemented validated scores for analgesia (6%, n = 3), for sedation (14%, n = 6) or for sedation and analgesia (79%, n = 35). ICUs which did not screen for delirium with validated scores, used sedation scores (21%, n = 12), pain scores (16%, n = 9), sedation and pain scores (42%, n = 24) or neither sedation or pain scores (21%, n = 12) (figure 2).

### Part two - Patients Data

In the second part of the survey ("part-two"), respondents of the first part were asked to enter patient specific data in a 32-item questionnaire.

**Figure 3:**
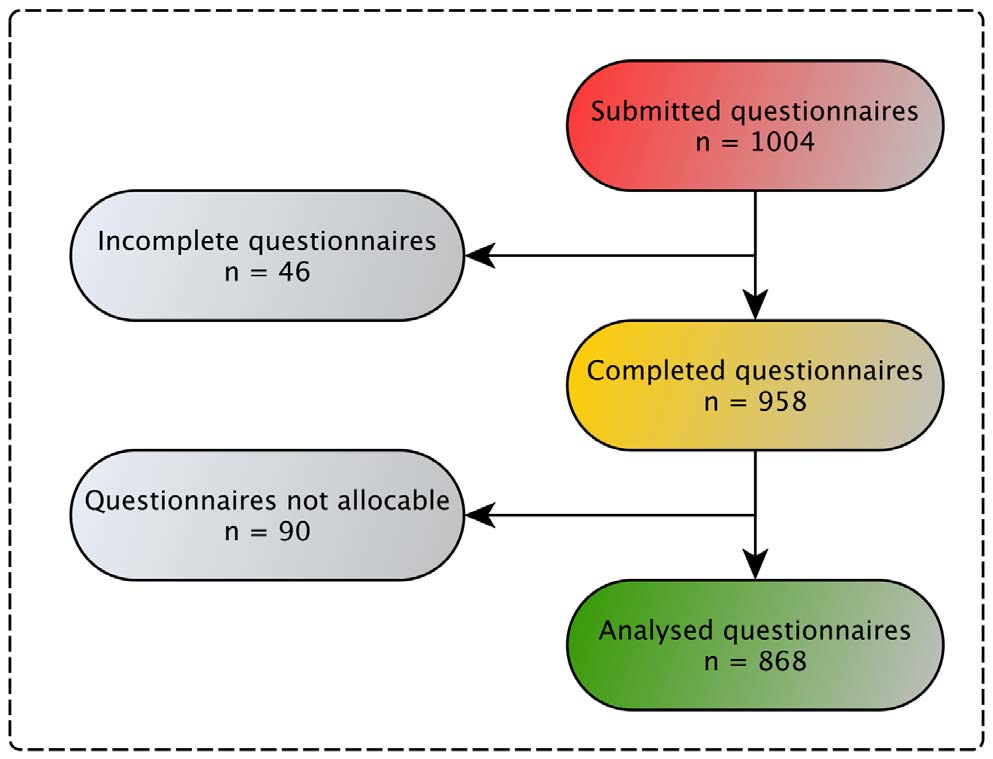
Consort diagram for questionnaire part 2. This part of the survey gathered actual practice on analgesia, delirium and sedation management among included patients. Not allocable  =  there was either no allocable token for questionnaire part one or most of the data were entered incorrectly (e.g. RASS  = 10, BPS  = −2).

One thousand and four questionnaires were submitted out of which 868 were fully completed and included in data analysis (figure 3). We summarized the charactaristics of patients in table 4.

**Table 4:** Demographic and clinical characteristics of included patients.

Characteristics (n = 868)	n (%)
Demographics	
Age, years	64 [51–73]*
Male	532 (61)
IMV	482 (56)
Type of admission	
Elective	238 (27)
Emergency	630 (73)
Reason for admission	
Surgical	378 (44)
Medical	440 (50)
Trauma	50 (6)

*Continuous variables are presented as medians with interquartile range [25th to 75th]. IMV, invasive mechanical ventilation.

The median time frame between ICU admission of patients and the day of study was 6 days (2–15). Respondents reported that 48% (n = 417) of the patients were monitored for symptoms of delirium; using a validated score in 27% (n = 234). Taking all patients into account that were evaluated with a validated delirium score, delirium prevalence was 44% (n = 103).

Fourty-three percent of patients (n = 369) were monitored with a validated sedation and pain score: 74% of them were were moderately or deeply sedated (RASS <−2/Ramsay>2/SAS <4) and 22% (n = 110) experienced significant pain levels (table 5).

**Table 5:** Methods and results of analgesia, delirium and sedation monitoring among included patients.

Symptom/Syndrome (n = 868)	n (%)
Analgesia	
No monitoring	499 (57)
Relevant pain*	110 (22)
Delirium	
No monitoring	451 (52.0)
Monitoring without score	183 (21.1)
Monitoring with score	234 (26.9)
Positive	103 (44.0)
Negative	131 (56.0)
Sedation	
No monitoring	499 (57)
Moderate to deep sedation**	273 (74)

Methods and results of monitoring that was actually performed in included patients (questionnaire part two). *NRS or VRS or VAS>4 or BPS>5. **RASS <−2 or Ramsay >2 or SAS <4. CAM-ICU, Confusion Assessment Method for the Intensive Care Unit. DDS, Delirium Detection Score. ICDSC, Intensive Care Delirium Screening Checklist.

## Discussion

Our data reveal an implementation rate of delirium assessment with a validated score of 44% which is to our knowledge the highest rate documented so far. However, "part two" of the survey revealed that only 27% of the patients were in fact monitored with a validated score.

Data from previous national and international surveys reported implementation rates between 2% and 34% [14]–[16]. Most of them were surveys on a national level. In-line with recently published surveys, more than half of the ICUs which participated in our survey belonged to a university hospital (table 1). Results of previous surveys indicate that routine delirium monitoring is more often performed in university hospitals than in small teaching or regional public hospitals [17]. This was confirmed by our data as 54% of the university hospitals used validated delirium scores compared to 29% of the non-academic hospitals. Furthermore, we observed that 11% of the respondents assessed for delirious symptoms without using a validated score. Literature suggests that delirium diagnosis due to clinical impression results in a high rate of failure or delayed detection [3].

In "part two" of the survey, we observed a one-day-prevalence of delirium of 44% which is consistent with previous reports [18]. Almost all of the ICUs (98%) stated that they treat delirious patients with specific pharmacological agents. Surprisingly, even ICUs that had not implemented some kind of delirium monitoring prescribed drugs for symptoms that maybe related to delirium. A national survey conducted by Gonçalves and colleagues revealed that delirium was the most frequent reason for sedation and midazolam was the most frequently used sedative [19]. There is increasing evidence that BZDs are a major risk factor for transitioning to delirium [20]. However, the majority of intensivists responded using BZDs for treatment of delirium.

In 2004 merely 8% of the ICUs in Germany monitored sedation with a validated score [21]. ICUs from our survey reported to use validated screening-instruments to assess sedation depth in 77% of the cases. The fact that only 43% of the patients were monitored regarding their sedation depth in actual practice emphasizes that structured training programs might be required to increase implementation rate [22].

If sedation is required, the coordination of a daily awakening and breathing trial has been shown to be an effective procedure to reduce mortality in ICU patients [23]. Thirty-four percent of the ICUs had implemented a paired SBT and SAT. These data reveal the necessity for improvement - especially regarding the implementation of evidence based sedation protocols.

From the patients' perspective, pain is the major stressors during critical illness [7], [24]. Results from previous studies in the field of pain management drew the conclusion that using specific instruments for assessment, significantly improved outcome in critically ill patients. [9], [25]. In our survey, eighty percent of respondents reported to routinely monitor for pain. Compared with results from previous surveys that showed implementation rates from 21% in general ICUs [26] up to 60% in European burn centers [15], our results indicate increasing awareness regarding pain assessment. A multicenter prospective observational study conducted in 2004/2005 revealed that specific pain scores were used in 28% of patients. Looking at our results of patient survey data, the use of pain scores drops to 43%. It is noteworthy that 70% of the ICUs did not use any assessment tool, specifically developed for assessing pain in sedated patients (e.g. Behavioural Pain Scale). Hence, sedated patients are at higher risk of suffering from insufficient analgesia.

This survey contributes to our knowledge about the management of analgesia, sedation and delirium in clinical practice. Until now, there are only two studies that have been conducted on an international level [15], [27]. Furthermore, this study is the first to report about delirium, sedation and analgesia in a general ICU population, taking into account differences between perceived and actual practice.

Nevertheless, limitations are inherent to surveys. We would expect a responder bias regarding the participants of this study. It is more likely that colleagues who are interested in delirium, sedation and analgesia participated in the survey. Taking this into account, it seems interesting that we found notable discrepancies between guideline recommendations and current practice. Considering the above mentioned responder bias, this observed gap may be even larger in reality. Further studies are necessary to evaluate this realtionship.

Despite an extensive preparation (technical support, email reminders etc.), a sixth of all webpage visitors completed the questionnaire.

Regional differences regarding the management of analgesia, delirium and sedation were not assessed.

The results of our survey indicate that awareness concerning a systematic management of delirium, sedation and analgesia and patient outcome is increasing. However, our data also show that the implementation of these measures in daily routine lacks behind. In our opinion, intelligent and sustainable implementation strategies are of key importance in order to transfer guideline recommendations to practise. The discrepancy between observed practice and perceived practice is significant and shows a wide gap between what we think we do and what we really do. Further studies will be necessary to assess and evaluate implementation strategies and improve clinical practice.

## Materials and Methods

This prospective, observational multicenter study (ethical approval No EA1/16- 5/10) was supported by the European Critical Care Research Network (ECCRN). The study was registered through Clinicaltrials.gov (identifier: NCT01278524) and approved by the data protection officer of the Charité - Universitaetsmedizin Berlin, Germany.

For collecting data, we developed two structured, web-based anonymous questionnaires in English (electronic case report file  =  eCRF) using the server based software LimeSurvey (Version 1.82+). The first questionnaire ("part one") contained 31 questions and was designed to gather general information about the participating hospital as well as (non-)pharmacological strategies for the management of analgesia, sedation and delirium (Appendix S1). In the second part, respondents were asked to enter patient specific data in a 32-item questionnaire. We asked to complete this questionnaire for each patient treated on the ICU during the 24 hour study period (January 25, 2011) (Appendix S2). In order to link hospital data of part one with patient related data of part two and keeping the survey anonymous, a token was generated for each participant: with opening the study website (www.improve-icu.com), users were presented a unique randomly generated numeric code. This code had to be entered at the beginning of each questionnaire. This numeric code allowed the allocation of the different questionnaires to one participating hospital.

We promoted the survey six months before it was carried out: Repeated email invitations were sent to all members of the European Society of Intensive Care Medicine (ESICM). Technical support was given by email and a telephone hotline on the day of study. Participants could access and complete the online questionnaires within one week. In addition to the online survey, participants were given the possibility to download a printable version of the survey and submit it by fax. Data from the printed and online questionnaires were merged in a database and exported to a worksheet for further statistical analysis.

Patients with a NRS or VRS or VAS of >4 or a BPS of >5 were considered to have relevant pain. Patients with a RASS <−2 or Ramsay >2 or SAS <4 werde considered to be moderately or deeply sedated.

Statistical Analysis: Exploratory data analysis were accomplished for all study variables. Discrete variables are expressed as counts (percentage) and continuous variables as medians with interquartile range (25th to 75th). Numerical calculations were performed with IBM SPSS Statistics Version 19 and Aabel 3.0.6, Gigawiz Ltd. Co.

## Supporting Information

Appendix S1 **First questionnaire (“part 1”).**
(PDF)

Appendix S2 **Second questionnaire (“part 2”).**
(PDF)

## References

[1] Pandharipande PP, Girard TD, Jackson JC, Morandi A, Thompson JL, et al (2013) Long-term cognitive impairment after critical illness. N Engl J Med 369: 1306–16. 24088092 10.1056/NEJMoa1301372 PMC3922401

[2] Ely EW, Shintani A, Truman B, Speroff T, Gordon SM, et al (2004) Delirium as a predictor of mortality in mechanically ventilated patients in the intensive care unit. JAMA 291: 1753–62. 15082703 10.1001/jama.291.14.1753

[3] Spronk PE, Riekerk B, Hofhuis J, Rommes JH (2009) Occurrence of delirium is severely underestimated in the icu during daily care. Intensive Care Med 35: 1276–80. 19350214 10.1007/s00134-009-1466-8 PMC2698979

[4] Devlin JW, Fong JJ, Schumaker G, O'Connor H, Ruthazer R, et al.. (2007) Use of a validated delirium assessment tool improves the ability of physicians to identify delirium in medical intensive care unit patients. Crit Care Med 35: : 2721–4; quiz 2725. 10.1097/01.ccm.0000292011.93074.82 18074477

[5] Devlin JW, Marquis F, Riker RR, Robbins T, Garpestad E, et al (2008) Combined didactic and scenario-based education improves the ability of intensive care unit staff to recognize delirium at the bedside. Crit Care 12: R19. 18291021 10.1186/cc6793 PMC2374631

[6] Shehabi Y, Chan L, Kadiman S, Alias A, Ismail WN, et al (2013) Sedation depth and long-term mortality in mechanically ventilated critically ill adults: a prospective longitudinal multicentre cohort study. Intensive Care Med 39: 910–8. 23344834 10.1007/s00134-013-2830-2 PMC3625407

[7] Pang PSK, Suen LKP (2008) Stressors in the icu: a comparison of patients' and nurses' perceptions. J Clin Nurs 17: 2681–9. 18808637 10.1111/j.1365-2702.2008.02280.x

[8] Battle CE, Lovett S, Hutchings H (2013) Chronic pain in survivors of critical illness: a retrospective analysis of incidence and risk factors. Crit Care 17: R101. 23718685 10.1186/cc12746 PMC4057262

[9] Payen JF, Bosson JL, Chanques G, Mantz J, Labarere J, et al (2009) Pain assessment is associated with decreased duration of mechanical ventilation in the intensive care unit: a post hoc analysis of the dolorea study. Anesthesiology 111: 1308–16. 19934877 10.1097/ALN.0b013e3181c0d4f0

[10] Martin J, Heymann A, Bäsell K, Baron R, Biniek R, et al (2010) Evidence and consensus-based german guidelines for the management of analgesia, sedation and delirium in intensive care–short version. Ger Med Sci 8: Doc02. 20200655 10.3205/000091 PMC2830566

[11] Young J, Murthy L, Westby M, Akunne A, O'Mahony R, et al (2010) Diagnosis, prevention, and management of delirium: summary of nice guidance. BMJ 341: c3704. 20667955 10.1136/bmj.c3704

[12] Barr J, Fraser GL, Puntillo K, Ely EW, Gélinas C, et al (2013) Clinical practice guidelines for the management of pain, agitation, and delirium in adult patients in the intensive care unit. Crit Care Med 41: 263–306. 23269131 10.1097/CCM.0b013e3182783b72

[13] Patel RP, Gambrell M, Speroff T, Scott TA, Pun BT, et al (2009) Delirium and sedation in the intensive care unit: survey of behaviors and attitudes of 1384 healthcare professionals. Crit Care Med 37: 825–32. 19237884 10.1097/CCM.0b013e31819b8608 PMC3719180

[14] Devlin JW, Bhat S, Roberts RJ, Skrobik Y (2011) Current perceptions and practices surrounding the recognition and treatment of delirium in the intensive care unit: a survey of 250 critical care pharmacists from eight states. Ann Pharmacother 45: 1217–29. 21934036 10.1345/aph.1Q332

[15] Trupkovic T, Kinn M, Kleinschmidt S (2011) Analgesia and sedation in the intensive care of burn patients: results of a european survey. J Intensive Care Med 26: 397–407. 21257635 10.1177/0885066610393442

[16] Forsgren LM, Eriksson M (2010) Delirium–awareness, observation and interventions in intensive care units: a national survey of swedish icu head nurses. Intensive Crit Care Nurs 26: 296–303. 20837322 10.1016/j.iccn.2010.07.003

[17] Van Eijk MMJ, Kesecioglu J, Slooter AJC (2008) Intensive care delirium monitoring and standardised treatment: a complete survey of dutch intensive care units. Intensive Crit Care Nurs 24: 218–21. 18524596 10.1016/j.iccn.2008.04.005

[18] Luetz A, Heymann A, Radtke FM, Chenitir C, Neuhaus U, et al (2010) Different assessment tools for intensive care unit delirium: which score to use? Crit Care Med 38: 409–18. 20029345 10.1097/CCM.0b013e3181cabb42

[19] Gonçalves F, Cordero A, Almeida A, Cruz A, Rocha C, et al (2012) A survey of the sedation practice of portuguese palliative care teams. Support Care Cancer 20: 3123–7. 22447339 10.1007/s00520-012-1442-7

[20] Pandharipande P, Cotton BA, Shintani A, Thompson J, Pun BT, et al (2008) Prevalence and risk factors for development of delirium in surgical and trauma intensive care unit patients. J Trauma 65: 34–41. 18580517 10.1097/TA.0b013e31814b2c4d PMC3773485

[21] Martin J, Parsch A, Franck M, Wernecke KD, Fischer M, et al (2005) Practice of sedation and analgesia in german intensive care units: results of a national survey. Crit Care 9: R117–23. 15774043 10.1186/cc3035 PMC1175921

[22] Radtke FM, Heymann A, Franck M, Maechler F, Drews T, et al (2012) How to implement monitoring tools for sedation, pain and delirium in the intensive care unit: an experimental cohort study. Intensive Care Med 38: 1974–81. 22945432 10.1007/s00134-012-2658-1

[23] Girard TD, Kress JP, Fuchs BD, Thomason JWW, Schweickert WD, et al (2008) Efficacy and safety of a paired sedation and ventilator weaning protocol for mechanically ventilated patients in intensive care (awakening and breathing controlled trial): a randomised controlled trial. Lancet 371: 126–34. 18191684 10.1016/S0140-6736(08)60105-1

[24] Novaes MA, Knobel E, Bork AM, Pavão OF, Nogueira-Martins LA, et al (1999) Stressors in icu: perception of the patient, relatives and health care team. Intensive Care Med 25: 1421–6. 10660851 10.1007/s001340051091

[25] Chanques G, Jaber S, Barbotte E, Violet S, Sebbane M, et al (2006) Impact of systematic evaluation of pain and agitation in an intensive care unit. Crit Care Med 34: 1691–9. 16625136 10.1097/01.CCM.0000218416.62457.56

[26] Martin J, Franck M, Sigel S, Weiss M, Spies C (2007) Changes in sedation management in german intensive care units between 2002 and 2006: a national follow-up survey. Crit Care 11: R124. 18062820 10.1186/cc6189 PMC2246220

[27] Shehabi Y, Botha JA, Boyle MS, Ernest D, Freebairn RC, et al (2008) Sedation and delirium in the intensive care unit: an australian and new zealand perspective. Anaesth Intensive Care 36: 570–8. 18714628 10.1177/0310057X0803600423

