# Supplementary material for: Delirium, Sedation and Analgesia in the Intensive Care Unit: A Multinational, Two-Part Survey among Intensivists
Source: PLoS One. 2014 Nov 14;9(11):e110935. doi: 10.1371/journal.pone.0110935 (PMC4232258; doi:10.1371/journal.pone.0110935)
Supplement: Appendix S1 — First questionnaire (“part 1”). (PDF) [file pone.0110935.s001.pdf]

IMPROVE-ICU Part I

There are 31 questions in this survey

General

1 Please enter your personal token *

Please write your answer here:

2 Hospital type *

Please choose **only one** of the following:

- University hospital
- Teaching hospital
- Other

3 Number of total hospital beds *

Please choose **only one** of the following:

- < 300
- 300 - 500
- > 500

4 Patients per year on ICU *

Please choose **only one** of the following:

- < 100
- 100 - 499
- 500 - 1499
- 1500 - 3000
- > 3000

5 ICU patients mainly *

Please choose **only one** of the following:

- Surgical
- Medical
- Mixed
- Other

6 ICU-beds *

Please write your answer(s) here:

Maximum	
Currently in use	

7 Are there beds for invasive / mechanically patients on your ICU? *

Please choose **only one** of the following:

- All beds are equipped for mechanical / invasive ventilation
- Some beds are equipped for mechanical / invasive ventilation, some not
- No beds are equipped for mechanical / invasive ventilation

8 Please specify number of beds for ventilated patients

[Only answer this question if you answered 'Some beds are equipped for mechanical / invasive ventilation, some not' to question '7']

Please write your answer here:

9 Intermediate care affiliated with the ICU if applicable

Please write your answer(s) here:

Maximum	
Currently in use	

10 Mean length of stay in ICU (days) *

Please choose **only one** of the following:

- ○ < 1
- ○ 1 - 3
- ○ 3 - 7
- ○ > 8
- ○ Information not available

11
Patients per staff during day shift

Please write your answer(s) here:

Patients per doctor	
Patients per nurse	

12 Scores used on your ICU *

Please choose **all** that apply:

- ☐ TISS 28
- ☐ APACHE II
- ☐ APACHE III
- ☐ SAPS
- ☐ SOFA
- ☐ None

Other: ____________

13 Is pain actively assessed and documented in your ICU? *

Please choose **only one** of the following:

- ○ Yes
- ○ No

14
Which pain scales are used in your ICU? *

[Only answer this question if you answered 'Yes' to question '13']

Please choose **all** that apply:

- ☐ Visual Analogue Scale (VAS)
- ☐ Numerical Analogue Scale (NAS)
- ☐ Behavioural Pain Scale (BPS)
- ☐ None

Other: ____________________

15 Please indicate frequency of pain monitoring. *

[Only answer this question if you answered 'Yes' to question '13']

Please choose **only one** of the following:

- ○ 2 hourly
- ○ 4 hourly
- ○ 6 hourly
- ○ 8 hourly
- ○ Hourly
- ○ Daily
- ○ As needed

16 Is sedation actively assessed and documented in your ICU? *

Please choose **only one** of the following:

- ○ Yes
- ○ No

17 Which sedation scales are in use on your ICU? *

[Only answer this question if you answered 'Yes' to question '16']

Please choose **all** that apply:

- ☐ RASS (Richmond Agitation Sedation Scale)
- ☐ SAS (Riker Sedation Agitation Scale)
- ☐ RSS (Ramsay Sedation Scale)
- ☐ None

Other: ____________________

18 At what interval is sedation monitored and documented on your ICU? *

[Only answer this question if you answered 'Yes' to question '16']

Please choose **only one** of the following:

- 1 hourly
- 2 hourly
- 4 hourly
- 8 hourly
- 12 hourly
- Daily
- Not monitored

Please indicated the answer that fits best.

19 Are daily sedation goals activelydefined and documented on your ICU? *

[Only answer this question if you answered 'Yes' to question '16']

Please choose **only one** of the following:

- Yes
- No
- Sometimes

20
Spontaneous breathing trials (SBT) in mecanically / invasive ventilated patients on your ICU are performed... *

[Only answer this question if you answered 'Yes' to question '16']

Please choose **only one** of the following:

- Never
- Daily
- Sometimes
- Not applicable (no mechanically / invasive ventilated patients on ICU)

21 Spontaneous awakening trials (SAT) in sedated patients on your ICU are performed... *

[Only answer this question if you answered 'Yes' to question '16']

Please choose **only one** of the following:

- ○ Never
- ○ Daily
- ○ Sometimes

22 How is sedation titrated on your ICU? *

[Only answer this question if you answered 'Yes' to question '16']

Please choose **all** that apply:

- ☐ Doctor's decision
- ☐ Nurse's decision
- ☐ Protocol driven
- ☐ Sedation is not titrated

Multiple answers possible

23 Is occurrence of delirium actively assessed and documented in your ICU? *

Please choose **only one** of the following:

- ○ Yes
- ○ No

24 Which delirium scores are in use on your ICU? *

[Only answer this question if you answered 'Yes' to question '23']

Please choose **all** that apply:

- ☐ None
- ☐ CAM (Confusion Assessment Method)
- ☐ CAM-ICU (The Confusion Assessment Method for the ICU)
- ☐ DDS (Delirium Detection Score)
- ☐ DRS-R98 (Delirium rating scale revised 98)
- ☐ ICDSC (Intensiv Care Delirium Screening Checklist)
- ☐ MDAS (Memorial Delirium Assessment Scale)
- ☐ Nu-DESC (Nursing Delirium Screening Scale)
- ☐ ICD-10 (International Classification of Diseases)
- ☐ DSM4 (diagnostical and statistical manual for mental disorders)

Other:

25 At which frequency is delirium screened and documented on your ICU? *

[Only answer this question if you answered 'Yes' to question '23']

Please choose **only one** of the following:

- 4 hourly
- 8 hourly
- 12 hourly
- 24 hourly

Please indicate the anwser that fits best.

Patients non-pharmacologic delirium measures

26 On your ICU: What sort of aids do you offer your patients to avoid delirium? *

Please choose **all** that apply:

- ☐ Hearing devices
- ☐ Glasses
- ☐ Encouring family involvement
- ☐ Mainting / restore the patient's body clock (sleep / wake cycle)
- ☐ No specific aids are offered

Other: ____________

27 On your ICU: Are special arrangements provided to remain or restore the patient's "body clock" (sleep-wake) (e.g. discourage naps, at night low-level lighting, quiet room)? *

Please choose **only one** of the following:

- ○ Yes, routinely
- ○ Yes, sometimes
- ○ No

28 Are delirious patient mobilized on your ICU ? *

Please choose **only one** of the following:

- ○ Yes
- ○ No
- ○ No delirium monitoring

29 Are physical restraints used in delirious patients on your ICU? *

Please choose **only one** of the following:

- ○ Yes, but only agitated patients
- ○ Yes, but only delirius patients
- ○ Yes, all ICU patients physically restrained
- ○ Physical restraining is never used

○ No delirium monitoring

30 On your ICU: What other measures are performed on regular base on delirious patients(please name)?

Please write your answer here:

Pharmacological delirium treatment on your ICU

31 What kind of pharmalogical treatment is used on your ICU?

Please choose **all** that apply:

- ☐ Antipsychotics (e.g. Haloperidol)
- ☐ Atypical antipsychotics (e.g. Risperidone, Olanzapine, Quetiapine)
- ☐ Benzodiazepines (e.g. Diazepam, Lorazepam)
- ☐ SARIs (e.g. Trazodone)
- ☐ Alpha-2 ag. (e.g. Clonidine, Dexmedetomidine)

Other: ____________

2010-08-03
{FAX_TO} Submit your survey.
Thank you for completing this survey.
