# Supplementary material for: Delirium, Sedation and Analgesia in the Intensive Care Unit: A Multinational, Two-Part Survey among Intensivists
Source: PLoS One. 2014 Nov 14;9(11):e110935. doi: 10.1371/journal.pone.0110935 (PMC4232258; doi:10.1371/journal.pone.0110935)
Supplement: Appendix S2 — Second questionnaire (“part 2”). (PDF) [file pone.0110935.s002.pdf]

# IMPROVE-ICU Part II

There are 32 questions in this survey

## Basic patient characteristics

### 1 Please enter your personal token *

Please write your answer here:

[ ]

### 2 Date of ICU admission *

Please enter a date:

[ ]

### 3 Please type of admission *

Please choose **only one** of the following:

- ○ Elective
- ○ Emergency

### 4 What was the initial reason for ICU admission? *

Please choose **only one** of the following:

- ○ Surgical
- ○ Medical
- ○ Trauma
- ○ Other [ ]

## 5 What was the mainly affected organ system upon ICU admission? *

Please choose **all** that apply:

- ☐ Central Nervous
- ☐ Cardiovascular
- ☐ Respiratory
- ☐ Gastrointestinal
- ☐ Genitourinary
- ☐ Trauma / Musculosceletal

Other:

## 6 Known disease of the central nervous system

[Only answer this question if you answered 'Central Nervous' to question '5' ]

Please choose **all** that apply:

- ☐ Dementia
- ☐ Stroke
- ☐ Traumatic brain injury

Other:

## 7 Dementia *

[Only answer this question if you answered 'Dementia' to question '6' ]

Please choose **only one** of the following:

- ○ Alzheimer's
- ○ Parkinson's
- ○ Other dementia

## 8 Patient’s age (years) *

Please write your answer here:

## 9 Patient's gender *

Please choose **only one** of the following:

- ○ Male
- ○ Female

## 10
## Respiratory support. Currently the patient is / receives ...
## *

Please choose **all** that apply:

- ☐ Not ventilated
- ☐ Non invasive ventilation (NIV)
- ☐ Invasive/mechanical ventilation
- ☐ Extracorporal lung assist

## 11 Duration (days) of mechanical / invasive ventilation

[Only answer this question if you answered 'Invasive/mechanical ventilation' to question '10' ]

Please write your answer here:

## 12 Duration (days) of extra corporal lung assist

[Only answer this question if you answered 'Extracorporal lung assist' to question '10' ]

Please write your answer here:

## 13 Devices

Please choose **all** that apply:

- [ ] Nasogastric tube
- [ ] Central venous line
- [ ] Intra-arterial line
- [ ] IABP
- [ ] Urinary catheter
- [ ] Dialysis catheter
- [ ] Epidural catheter
- [ ] ICP monitor

Other: ____________

## 14 Drains (number)

Please write your answer here:

____________

# Patients Score Values (last 24h)

**Please type 999 for not applicable**

## 15 24 hours before day of study (January 24th, 00:00:00-23:59:59)

## 16 TISS 28

Please write your answer here:

## 17 APACHE II

Please write your answer here:

## 18 APACHE III

Please write your answer here:

## 19 SAPS

Please write your answer here:

## 20 SOFA

Please write your answer here:

## 21 Others (which)

**Please write your answer here:**

# Patient’s Pain Data

## 22 What analgesics has the patient received during the 24 hours preceding the day of study? *

**Please choose all that apply:**

- ☐ None used
- ☐ Fentanyl
- ☐ Sufentanil
- ☐ Remifentanil
- ☐ Morphine
- ☐ Piritramide
- ☐ epidural

**Other:** [ ]

**24 hours before day of study (January 24th, 00:00:00-23:59:59)**

## 23
## Patients highest pain score (score)

| | VAS | NAS | BPS | Other |
|---|---|---|---|---|
| Morning shift | | | | |
| Afternoon shift | | | | |
| Night shift | | | | |

**24 hours before day of study (January 24th, 00:00:00-23:59:59)**

**For each shift, please indicate only the highest score that has been documented and leave the others blank.**

## 24
## If you have used the column "other", please specify the name of the score

**Please write your answer here:**

[ ]

# Patient's Sedation Data

## 25 What sedatives has the patient received during the 24 hours preceding the day of study? *

**Please choose all that apply:**

- ☐ None used
- ☐ Propofol
- ☐ Midazolam
- ☐ Lorazepam
- ☐ Clonidine
- ☐ Dexmedetomidine
- ☐ Ketamine
- ☐ Barbiturates

**Other:** ______

**24 hours before day of study (January 24th, 00:00:00-23:59:59)**

## 26 Please name barbiturates

**[Only answer this question if you answered 'Barbiturates' to question '25' ]**

**Please write your answer here:**

______

## 27 Sedation scale (score)

**[Only answer this question if you have NOT answered 'None used' to question '25' ]**

| | RASS | SAS | RSS | Other |
|---|---|---|---|---|
| Morning shift (pts) | | | | |
| Afternoon shift (pts) | | | | |
| Night shift (pts) | | | | |

**24 hours before day of study (January 24th, 00:00:00-23:59:59)**

**For each shift, please indicate only the highest score that has been documented and leave the others blank.**

## 28
## If you have used the column "other", please specify the name of the score

**Please write your answer here:**

# Patient's Delirium Data

**29**

**Was the patient delirious during the 24 hours preceding the day of study? ***

**Please choose only one of the following:**

- ○ Yes
- ○ No
- ○ Delirium not monitored
- ○ Evaluation not possible (e.g. RASS <= -3, GCS 3)

**24 hours before day of study (January 24th, 00:00:00-23:59:59)**

**30**

**By whom was occurence or non-occurence of delirium detected? ***

**[Only answer this question if you answered 'Yes' or 'No' to question '29' ]**

**Please choose only one of the following:**

- ○ Nurse
- ○ Intensive Care MD
- ○ Psychiatrist
- ○ Psychologist
- ○ Other ______

## 31
## Which score/method was used for detection?

**[Only answer this question if you answered 'No' or 'Yes' to question '29' ]**

**Please choose all that apply:**

- ☐ None
- ☐ CAM (Confusion Assessment Method)
- ☐ CAM-ICU (The Confusion Assessment Method for the ICU)
- ☐ DDS (Delirium Detection Score)
- ☐ DRS-R98 (Delirium rating scale-revised-98)
- ☐ ICDSC (Intensive Care Delirium Screening Checklist)
- ☐ MDAS (Memorial Delirium Assessment Scale)
- ☐ Nu-DESC (Nursing Delirium Screening Scale)
- ☐ ICD-10 (International Classification of Diseases)
- ☐ DSM4 (diagnostical and statistical manual for mental disorders)

**Other:** ____________

## 32
## Patients highest delirium score (score)

**[Only answer this question if you answered 'Yes' or 'No' to question '29' ]**

**Please write your answer(s) here:**

| | |
|---|---|
| **Morning shift** | |
| **Afternoon shift** | |
| **Night shift** | |

**24 hours before day of study (January 24th, 00:00:00-23:59:59)**
**If score result is "positive" or "negative" (e.g. CAM-ICU), please type 100 for positive and 200 for negative**

**2010-08-04**
**{FAX_TO} Submit your survey.**
**Thank you for completing this survey.**
